# Spin-Valve-Controlled Triggering of Superconductivity

**DOI:** 10.3390/nano14030245

**Published:** 2024-01-23

**Authors:** Alexey Neilo, Sergey Bakurskiy, Nikolay Klenov, Igor Soloviev, Mikhail Kupriyanov

**Affiliations:** 1 Skobeltsyn Institute of Nuclear Physics, Lomonosov Moscow State University, Moscow 119991, Russia; aleks.neilo@yandex.ru (A.N.); r3zz@mail.ru (S.B.); mkupr@pn.sinp.msu.ru (M.K.); 2 National University of Science and Technology MISIS, Moscow 119049, Russia; nvklenov@mail.ru; 3 Dukhov All-Russia Research Institute of Automatics, Moscow 101000, Russia; 4 Faculty of Physics, Moscow State University, Moscow 119991, Russia

**Keywords:** proximity effect, multilayered structures, superconducting spin valve

## Abstract

We have studied the proximity effect in an SF_1_S_1_F_2_s superconducting spin valve consisting of a massive superconducting electrode (S) and a multilayer structure formed by thin ferromagnetic (F_1,2_) and superconducting (S_1_, s) layers. Within the framework of the Usadel equations, we have shown that changing the mutual orientation of the magnetization vectors of the F_1,2_ layers from parallel to antiparallel serves to trigger superconductivity in the outer thin s-film. We studied the changes in the pair potential in the outer s-film and found the regions of parameters with a significant spin-valve effect. The strongest effect occurs in the region of parameters where the pair-potential sign is changed in the parallel state. This feature reveals new ways to design devices with highly tunable inductance and critical current.

## 1. Introduction

The development of low-dissipation and non-volatile memory and control elements is one of the main tasks in superconducting electronics [1,2,3,4,5,6,7,8,9,10,11]. These elements can significantly help in the design of supercomputers, data centers, neuromorphic circuits, and quantum computing. The use of hybrid structures consisting of superconductors (S) and ferromagnets (F) is one of the modern and promising areas in the development of these devices [12,13,14,15,16,17,18,19,20,21,22].

The interaction between superconducting and ferromagnetic orders in SF structures can lead to the appearance of a number of peculiar effects, opening up the prospect of creating effective superconducting spin valves (SSVs) [23,24,25,26,27]. Depending on the physical parameter being controlled, SSVs can be divided into three types. These are devices in which a change in the mutual orientation of the magnetic moments of the F-films is accompanied by a variation in their critical current [5,24,27,28,29,30,31,32,33,34,35,36,37,38,39], critical temperature [26,40,41,42,43,44,45], or kinetic inductance [4,8,19].

**Figure 1:**
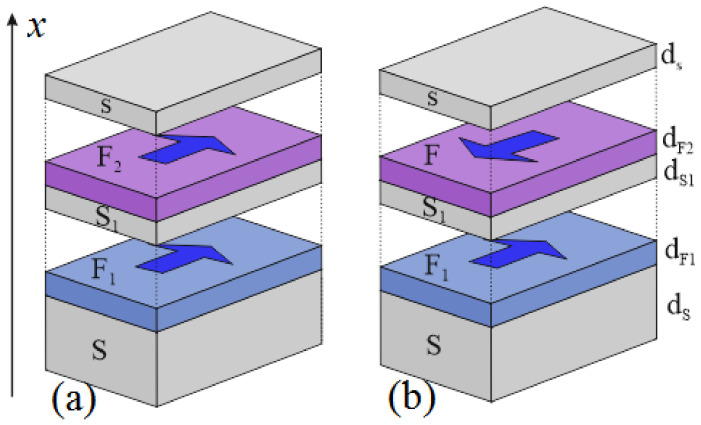
Sketch of the SF_1_S_1_F_2_s structure in P (**a**) and AP (**b**) orientations of magnetization. Note that the upper layer can be transferred from the superconducting state to the normal state and vice versa by changing the mutual orientation of the magnetization vectors of the ferromagnetic layers of the structure.

At present, the last type of the above-mentioned spin valves is the least studied among the variety of possible technical solutions. In contrast to the other types of tunable inductors [17,46,47], it does not require the current suppression of superconductivity and can be considered a tunable linear element. The typical configuration of such a device is shown in Figure 1. It consists of a massive S-electrode and an F_1_S_1_F_2_s multilayer structure formed by thin ferromagnetic (F_1,2_) and superconducting (S_1_, s) layers. Superconductivity in the outer s-film of the SF_1_S_1_F_2_s structure is maintained by both intrinsic superconducting correlations and the proximity effect of the massive S-electrode. The intensity of these sources of superconductivity and, consequently, the order parameter in the outer s-film $\Delta_{s}$ , as well as the kinetic inductance of the structure, are determined by the mutual orientation of the magnetization vectors of its F-layers. It is supposed that the presence of the bulk S-electrode leads to an increase (compared to F_1_S_1_F_2_s spin valves) in the difference in the magnitude of $\Delta_{s}$ for the parallel (P) and antiparallel (AP) orientations of the magnetization vectors. However, quantitative estimates of the maximum magnitude of the possible spin-valve effect is still to be obtained. The ranges of the SF_1_S_1_F_2_s structural material parameters where this maximum is reached are also unknown.

The aim of this work is to verify this conjecture by formulating the criteria for the structure to exhibit a potent spin-valve effect and to find the set of material constants that would allow the selection of suitable materials for the design of SF_1_S_1_F_2_s SSVs.

## 2. Model

We assume that the conditions of the dirty limit are satisfied for all the films in the SF_1_S_1_F_2_s multilayer. We also restrict ourselves to considering only the parallel and antiparallel orientations of F-film magnetization vectors $M_{1,2}$ .

Under these conditions, we can study the proximity problem in the SF_1_S_1_F_2_s SSV in the framework of the one-dimensional Usadel equations [48] with Kupriyanov–Lukichev boundary conditions [49] at the SF and Fs interfaces.

(1) $$\frac{\pi T_{C}\xi_{p}^{2}}{{\tilde{\omega }}_{p}G_{m}}\frac{d}{dx}\left(G_{p}^{2}\frac{d\Phi_{p}}{dx}\right)-\Phi_{p}=-\Delta_{p}$$

(2) $$\Delta_{p}\ln\frac{T}{T_{C}}+\frac{T}{T_{C}}\sum_{\omega =-\infty }^{\infty }\left(\frac{\Delta_{p}}{\left|\omega \right|}-\frac{\Phi_{p}G_{p}}{\omega }\right)=0,$$

(3) $$\pm \gamma_{Bpq}\xi_{p}G_{p}\frac{d}{dx}\Phi_{p}=G_{q}\left(\frac{{\tilde{\omega }}_{p}}{{\tilde{\omega }}_{q}}\Phi_{q}-\Phi_{p}\right).$$

In Equations (1)–(3), *p* and *q* are the subscripts of the corresponding layers, $G_{p}={\tilde{\omega }}_{p}/\sqrt{{\tilde{\omega }}_{p}^{2}+\Phi_{p,\omega }\Phi_{p,-\omega }^{*}}$ and ${\tilde{\omega }}_{p}=\omega +iH_{p}$ , ω=πT(2n+1) are Matsubara frequencies, $\Delta_{p}$ is the pair potential, $H_{p}$ is the exchange energy of the ferromagnetic layer ( $H_{p}=0$ in nonferromagnetic materials), $T_{C}$ is the critical temperature of the bulk superconductor, $\xi_{p}={\left(D_{p}/2\pi T_{C}\right)}^{1/2}$ is the coherence length, $D_{p}$ is the diffusion coefficient, $G_{p}$ and $\Phi_{p}$ are normal and anomalous Green’s functions, respectively, $\gamma_{Bpq}=R_{Bpq}A_{Bpq}/\rho_{p}\xi_{p}$ is a suppression parameter, $R_{Bpq}$ and $A_{Bpq}$ are the resistance and area of the corresponding interface, and $\rho_{p}$ is the resistivity of the *p*-th film. The plus sign in (3) means that the *p*-th material is located on the side $x_{m}-0$ from the interface position $x_{m}$ , and the minus sign corresponds to the case where the *p*-th material is at $x_{m}+0$ . Hereafter, we use the following normalization: ℏ=1 and $k_{B}=1$ . The boundary conditions at free interfaces, ∂Φ/∂n=0 , follow from the the requirement that there be no current across them. Here, *n* is the direction of the normal to the corresponding boundary.

Below, we characterize the degree of superconducting correlations in the outer s-film by the magnitude of the order parameter $\Delta_{s}$ at its free surface and by the difference, $\delta =\left|\Delta_{↑↓}\right|-\left|\Delta_{↑↑}\right|,$ in the $\Delta_{s}$ values calculated in the antiparallel ( $\left|\Delta_{↑↓}\right|$ ) and parallel ( $\left|\Delta_{↑↑}\right|$ ) directions of the F-layer magnetization vectors.

The formulated boundary-value problem (1)–(3) has been solved numerically [50]. We set the temperature $T=0.5T_{C}$ and the thickness of the thick S-layer $d_{S}=5\xi_{S}$ . We also used the exchange energy $H_{p}=100T_{C}$ and a suppression parameter $\gamma_{B}=0.3$ for both F-films and for all FS boundaries. These parameters are typical of Nb interfaces with ferromagnetic alloys (see the review in [51] and references therein), with a liquid helium working temperature and a $T_{C}$ of about 9 K for Nb. The boundary-value problem (1)–(3) was solved using numerical methods developed for solutions to nonlinear differential equations through LU factorization for band matrices with three diagonals, in combination with the relaxation method [50]. The numerical algorithm has been adapted to solve the Usadel equations, where the superconducting order parameter is treated as a given coordinate function. The exit from the iterative loop on nonlinearities occurred when the difference between two successive iterations reached an accuracy of $10^{-9}$ . The anomalous Green’s functions thus computed were then used to compute a new coordinate dependence of the order parameter. The resulting dependence Δ(x) was again substituted into the Usadel equations. The exit from the iteration cycle by Δ(x) was realized when the maximum difference between two successive iterations was less than $10^{-6}T_{C}$ .

## 3. Proximity Effect in SF_1_S_1_F_2_s Trigger

We begin our analysis by studying the proximity effect in an SF_1_S_1_F_2_s SSV when the resistivities of all materials in the structure ( $\rho_{F}=\rho_{S}$ ) are the same, the coherence lengths $\xi_{F1}=\xi_{F2}$ , and the thicknesses of the S- and F-layers are equal to $d_{F1}=0.15\xi_{S}$ , $d_{S1}=0.2\xi_{S}$ , and $d_{F2}=0.25\xi_{S}$ .

**Figure 2:**
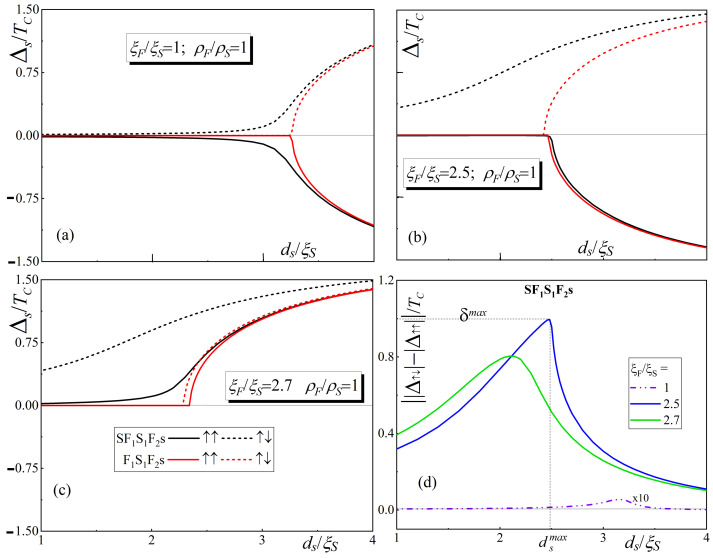
Dependence of the order parameter on the s-layer free surface $\Delta_{s}$ (panels (**a**)–(**c**)) as a function of $d_{s}$ in the case of P and AP orientations of the vectors $M_{1,2}$ (solid and dotted lines, respectively) for SF_1_S_1_F_2_s and F_1_S_1_F_2_s structures (black and red colors, respectively). The curves are calculated for three values of the parameter $\xi_{F}/\xi_{S}$ , equal to 1, 2.5 , and 2.7 ((**a**), (**c**), and (**d**) panels, respectively). Dependence of the parameter δ (panel (**d**)) on $d_{s}$ for values of the parameter $\xi_{F}/\xi_{S}$ equal to 1, 2.5 , and 2.7 (black, red, and blue colors, respectively). In panel d, the values of δ , calculated with $\xi_{F}/\xi_{S}=1$ , are increased by a factor of 10 for the sake of clarity. The other parameters of the SF_1_S_1_F_2_s-(F_1_S_1_F_2_s-) structure are $d_{S}=5\xi_{S}$ , $d_{F1}=0.15\xi_{S}$ , $d_{S1}=0.2\xi_{S}$ , $d_{F2}=0.25\xi_{S}$ , $H=100T_{C}$ , $T=0.5T_{C}$ , $\gamma_{B}=0.3$ , $\rho_{F}=\rho_{S}$ , $\xi_{F1}=\xi_{F2}$ .

Figure 2a–c show the dependencies of the order parameter on the free surface of the s-layer $\Delta_{s}$ and the parameter δ (panel d) on its thickness $d_{s}$ in the case of P (dotted lines) and AP orientations (solid lines) of the magnetization vectors $M_{1,2}$ for the SF_1_S_1_F_2_s and F_1_S_1_F_2_s structures without a bottom superconductor electrode. The curves are calculated for different $\xi_{F}/\xi_{S}$ ratios, equal to 1, 2.5 , and 2.7 . As expected, the transition from the parallel to antiparallel mutual orientation of the vectors $M_{1,2}$ is accompanied by an increase in the magnitude of the modulus of the order parameter $\Delta_{s}$ on the free surface of the s-film. Note that for $\xi_{F}/\xi_{S}=1$ and $\xi_{F}/\xi_{S}=2.5$ , the switching is accompanied by a change in the sign of the order parameter.

First of all, it should be emphasized that an increase in the ratio $\xi_{F}/\xi_{S}$ actually means a decrease in the thickness of the F-films in units of $\xi_{F}$ . This is the reason for the observed growth of $\Delta_{s}\left(d_{s}\right)$ with an increase in $\xi_{F}/\xi_{S}$ at a fixed value of $d_{s}/\xi_{S}$ .

The calculations show that, for the largest effective thickness of the F-layers ( $\xi_{F}/\xi_{S}=1$ ) in the F_1_S_1_F_2_s multilayer, the superconducting correlations are completely suppressed at $d_{s}=d_{cr}\approx 3.4\xi_{S}$ (for the case $\xi_{F}/\xi_{S}=1$ ), and $d_{cr}$ practically does not depend on the mutual orientation of the vectors $M_{1,2}$ . This independence is preserved even at smaller thicknesses of the F-layers, which is confirmed by calculations at $\xi_{F}/\xi_{S}=2.5$ and $\xi_{F}/\xi_{S}=2.7$ with $d_{cr}\approx 2.5\xi_{S}$ and $d_{cr}\approx 2.4\xi_{S}$ , respectively. This means that there is no standard spin-valve effect in the structure associated with a change in the effective exchange energy in the ferromagnetic part of the device acting on the s-superconductor. In other words, the superconductivity in the s-layer depends only on the proximity effect of the F_2_-film.

The situation in SF_1_S_1_F_2_s devices is completely different. The presence of a massive superconducting S-electrode, whose weakest point is moved closer to the center of the structure, gives additional support to the superconductivity in the s-film. This can be seen from the shape of the black curves in Figure 2a: the magnitude of $\left|\Delta (d_{s})\right|$ at $\xi_{F}/\xi_{S}=1$ and $d_{s}=d_{cr}$ shifts from 0 up to $\approx 0.5T_{c}$ , and the dependence drops more smoothly to 0 after $d_{s}<d_{cr}$ .

It is important to note that for a given $d_{s}$ , the reversal of the direction of the magnetization vector of one of the F-layers to the opposite direction is accompanied by a change in the sign of $\Delta_{s}$ , keeping the difference δ at a negligibly small level. This means that the thickness of the F_2_-film appears to be so large that the additional superconducting support provided by the S-layer practically does not reach the s-film and only provides a phase shift between the superconducting correlations in the S- and s-parts of the SF_1_S_1_F_2_s structure. Note that in the F_2_s proximity system, the magnitude of $\Delta_{s}$ does not depend on the phase of the correlation leading to δ=0 . The small deviation of δ from zero found as a result of the calculations is due to the effect of proximity between the SF_1_S_1_ and F_2_s parts of the SF_1_S_1_F_2_s structure.

A decrease in the effective thickness of the ferromagnetic layers is accompanied by an increase in the mutual influence of the SF_1_S_1_F_2_ and F_2_s blocks. Figure 2b,c show that at $\xi_{F}/\xi_{S}=2.5$ and $\xi_{F}/\xi_{S}=2.7$ , there is a significant increase in the absolute values of δ . It is seen in Figure 2d that the dependence of $\Delta_{s}$ is a nonmonotonic function of the s-layer thickness. It achieves a maximum at $d_{s}=d_{s}^{max}\approx d_{cr}$ .

With $d_{s}\leq d_{cr}$ and the parallel orientation of the vectors $M_{1,2}$ , the superconductivity in the s-layer turns out to be almost completely suppressed and weakly dependent on $d_{s}$ . In this thickness region, the observed growth of δ with increasing $d_{s}$ is due to an increase in the superconductivity induced in the s-layer, which occurs in the AP configuration of the vectors $M_{1,2}$ .

At $d_{s}>d_{cr}$ , there is intrinsic superconductivity in the s-film. This is manifested by the growth of the $\Delta_{s}$ module with increasing $d_{s}$ and a monotonous decrease in the dependence of $\Delta_{s}$ . The larger $d_{s}$ is, the stronger the intrinsic superconductivity is in the s-film and the closer $\Delta_{s}$ is to zero.

**Figure 3:**
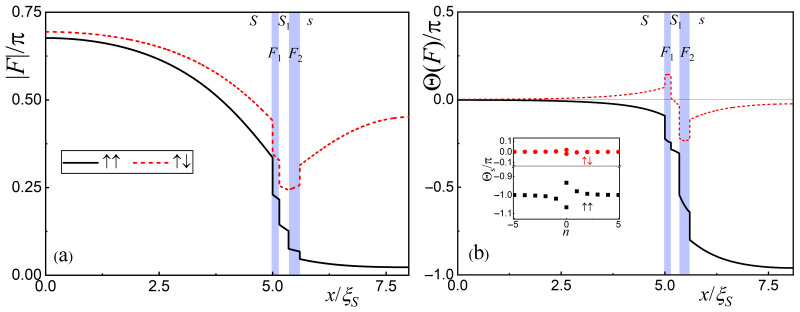
Spatial distributions of module of the pair amplitudes *F* and their phases Θ at the first Matsubara frequency (panels (**a**) and (**b**), respectively), calculated for $\xi_{F}/\xi_{S}=2.5$ , $d_{s}=2.5\xi_{S}$ , and P and AP orientations (black solid and red dotted lines, respectively). The blue rectangles indicate the areas occupied by the ferromagnetic layers. The inset in panel (**b**) shows the phase value on the free *s*-surface $\Theta_{s}$ for different Matsubara frequencies. The other parameters of the SF_1_S_1_F_2_s structure are $d_{S}=5\xi_{S}$ , $d_{F1}=0.15\xi_{S}$ , $d_{S1}=0.2\xi_{S}$ , $d_{F2}=0.25\xi_{S}$ , $H=100T_{C}$ , $T=0.5T_{C}$ , $\gamma_{B}=0.3$ , $\rho_{F}=\rho_{S}$ , $\xi_{F1}=\xi_{F2}$ .

Figure 3 provides a deeper insight into the characteristics of the proximity effect in the SF_1_S_1_F_2_s structure. The graphs demonstrate the spatial distribution of the module of the pair amplitude $F\left(x\right)=\Phi_{p,\omega }/\sqrt{{\tilde{\omega }}_{p}^{2}+\Phi_{p,\omega }\Phi_{p,-\omega }^{*}}$ (panel a) and its phase $\Theta =\arctan\left(Im(F)/Re(F)\right)$ (panel b) calculated for the first Matsubara frequency and $\xi_{F}/\xi_{S}=2.5$ , $d_{s}=d_{s}^{max}=2.5\xi_{S}$ . The results obtained with the parallel and antiparallel orientations of the F-film magnetization vectors are shown as solid black and dashed red curves, respectively. The blue rectangles indicate the areas occupied by the ferromagnetic layers.

It can be seen that the presented curves are qualitatively different from similar dependencies characterizing the proximity effect in SN and SF multilayer structures with a ferromagnetic film on the free surface. In the SN multilayer case, there should also be jumps in the F-module at the interfaces. However, these jumps do not lead to a change in the phase of the F(x) functions. It does not depend on the spatial coordinate and must coincide with the phase of the massive S-electrode.

In multilayer SF structures, the decay of superconducting correlations in F-layers has a damping oscillatory character. This feature causes both module and phase jumps of anomalous functions to occur at the interfaces. Figure 3 shows that the amplitudes of these jumps can differ between adjacent boundaries. The condition that the normal derivative of the anomalous functions *F* is equal to zero selects, from all their possible spatial configurations, only those that provide an extremum of *F* on the outer surface of the F-layer. Such phase synchronization leads to the fact that, among all possible spatial configurations F(x) , only those in which the phase difference between the massive S-electrode and the outer F-layer is equal to either 0 or π are realized.

The SF_1_S_1_F_2_s structure we are studying ends with an s-film in which the spatial variations are not oscillatory. In this case, the jumps of the module and the phase of the functions *F* on the internal interfaces impose a spatial dependence Θ(x) , which ensures the absence of a current in the multilayer. For this reason, the values of $\Theta (d_{s})$ in Figure 3b are slightly different from 0 in the AP state and are not equal to π with the parallel orientation of the magnetization vectors for the 1-st Matsubara frequency and rather quickly converges to π as their number increases.

We can draw three important conclusions from our analysis of the proximity effect.

First, in the SF_1_S_1_F_2_s structure, there is a phase mismatch of anomalous Green functions on the free surface of the s-film. They do not coincide with each other and do not match the phase of the order parameter. This problem should be taken into account when designing any device containing such a structure as an electrode in a multilayer tunnel junction [52] or as a kinetic inductor in detectors or neuromorphic circuits [19].

Second, we have shown that with $\xi_{F}/\xi_{S}=2.5$ and the fixed values of the other parameters of the studied structure, a significant spin-switching effect is realized at the thickness of the s-layer $d_{s}\approx d_{cr}$ . Namely, the switching of the mutual orientation of the magnetization vectors of the F-layers is accompanied by a change in the magnitude of the parameter modulus from values close to zero to values comparable to the values of Δ in a massive S-electrode.

Third, a significant difference between $d_{cr}^{P}$ and $d_{cr}^{AP}$ proves the possibility of using the standard SF_1_S_1_F_2_ spin valve not only for standard S-layer superconductivity control operations but also as a tool to switch superconductivity on or off in the F_2_s part of a structure weakly coupled to the SF_1_S_1_F_2_ spin valve. Thus, the SF_1_S_1_F_2_ spin valve actually performs the function of a trigger that turns superconductivity on or off in the F_2_s part of the SF_1_S_1_F_2_s device.

In the following, we will analyze how stable the obtained trigger effect (TE) is by examining the dependence of the maximum achievable value $\delta \left(d_{s}\right)=\delta^{max}$ and the thickness of the s-layer $d_{s}=d_{s}^{max}$ at which this maximum is reached on the material and geometrical parameters of the SF_1_S_1_F_2_s structure.

## 4. Influence of Material Properties and Structural Dimensions on the Trigger Effect

**Figure 4:**
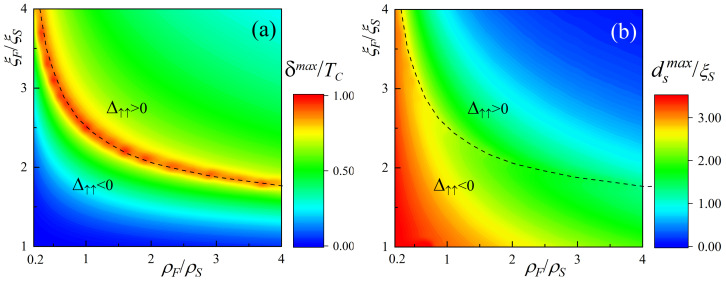
Maps of material parameters of ferromagnets $\rho_{F}$ , $\xi_{F}$ for the maximum difference during magnetization reversal $\delta^{max}$ (**a**) and achieved at thicknesses $d_{s}^{max}$ (**b**). Below the dotted line in the P orientation, the Δ in the s-layer is negative; above the line, it is positive. The other parameters of the SF_1_S_1_F_2_s structure are $d_{S}=5\xi_{S}$ , $d_{F1}=0.15\xi_{S}$ , $d_{S1}=0.2\xi_{S}$ , $d_{F2}=0.25\xi_{S}$ , $H=100T_{C}$ , $T=0.5T_{C}$ , $\gamma_{B}=0.3$ .

The conclusions formulated in the previous section were based on calculations performed for $\rho_{F}=\rho_{S}$ and three fixed ratios of $\xi_{F}/\xi_{S}$ . To understand how stable they are with respect to the variation in these ratios, we generated the maps shown in Figure 4a,b. The values of all the other parameters remained the same as in the calculation of the curves shown in Figure 2.

In Figure 4a, the color palette shows the values of the parameter $\delta^{max}$ as a function of the $\rho_{F}/\rho_{S}$ and $\xi_{F}/\xi_{S}$ ratios. The red color corresponds to the maximum values of $\delta^{max}$ . The blue color corresponds to the minimum values. The dashed curve divides the plane of the parameters $\rho_{F}/\rho_{S}$ and $\xi_{F}/\xi_{S}$ into two regions. In the upper-right corner above this curve, the values $\Delta_{s}$ are positive. Below this curve they are negative.

It can be seen that in the vicinity of $\xi_{F}/\xi_{S}\approx 1$ (blue area in the lower part of Figure 4a), the values of $\delta^{max}$ are close to zero, regardless of the ratio of $\rho_{F}/\rho_{S}$ . At $\xi_{F}/\xi_{S}≳1.8$ , there is a noticeable trigger effect ( $\delta^{max}/T_{C}≳0.5$ ) at almost any ratio of $\rho_{F}/\rho_{S}$ . The strongest triggering effect $\delta^{max}/T_{C}\approx 1$ occurs in the region shown by the dashed line, where the value of $\Delta_{P}$ changes its sign.

The second important parameter in Figure 2 is the thickness of the s-film $d_{s}^{max}$ at which the trigger effect is maximal. In Figure 4b, the color palette shows the values of the parameter $d_{s}^{max}$ as a function of the the $\rho_{F}/\rho_{S}$ and $\xi_{F}/\xi_{S}$ ratios. The red color corresponds to the maximum values of $d_{s}^{max}$ . The blue color corresponds to the minimum values. The data presented in Figure 4b allow us to determine, for fixed values of $\rho_{F}/\rho_{S}$ , $\xi_{F}/\xi_{S}$ , and $\delta^{max}$ , which thickness of the s-layer should be chosen to produce the state with the maximum TE. It should be noted that although the maximum $\delta_{max}$ amplitude occurs in a wide range of parameters, the corresponding $d_{s}^{max}$ is different at different points. For example, the maximum TE occurs for $\rho_{F}=0.3\rho_{S}$ , $\xi_{F}=4\xi_{S}$ at $d_{s}^{max}\approx 3\xi_{S}$ , while the same TE for $\rho_{F}=4\rho_{S}$ , $\xi_{F}=1.8\xi_{S}$ is realized at $d_{s}^{max}\approx 1\xi_{S}$ . In the design of small-scale superconducting devices using a trigger effect in control elements, this feature can be important and useful.

**Figure 5:**
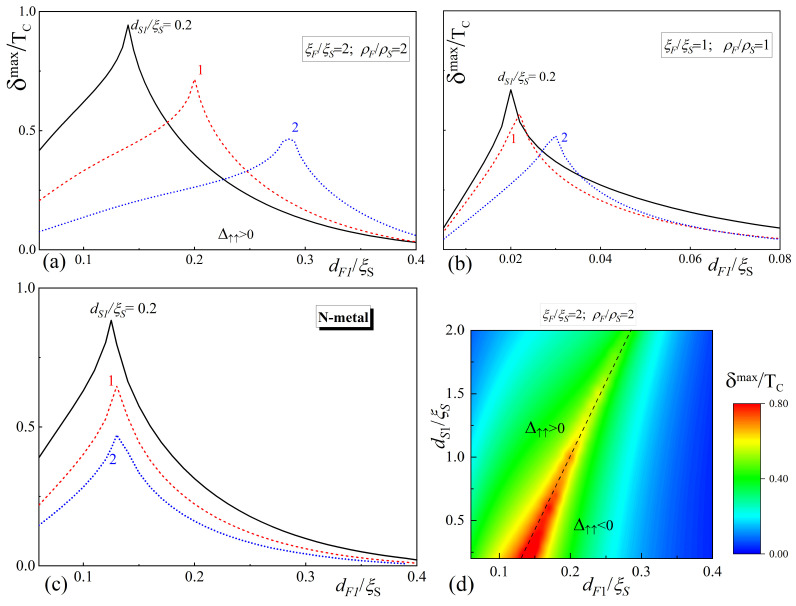
Dependence of the maximum difference $\delta^{max}$ upon magnetization reversal on the thickness of ferromagnets $d_{F1}$ for the material parameters $\rho_{F}/\rho_{S}=2$ , $\xi_{F}/\xi_{S}=2$ (**a**) and $\rho_{F}/\rho_{S}=1$ , $\xi_{F}/\xi_{S}=1$ (**b**) and for the case when the middle layer is a normal metal (**c**). Panel (**d**) is the $\delta^{max}$ map depending on the thicknesses of the ferromagnets $d_{F1}$ and the thickness of the superconducting middle layer $d_{S1}$ . In the calculations, it was always assumed that $d_{F2}=d_{F1}+0.1\xi_{S}$ . The other parameters of the SF_1_S_1_F_2_s(SF_1_NF_2_s) structure were $d_{S}=5\xi_{S}$ , $d_{S1}=0.2\xi_{S}$ , $H=100T_{C}$ , $T=0.5T_{C}$ , $\gamma_{B}=0.3$ .

To evaluate the influence of geometric factors on the TE effect, we set $d_{F2}=d_{F1}+0.1\xi_{S}$ and examined the dependence of $\delta^{max}$ on $d_{F1}/\xi_{S}$ for S_1_-layer thicknesses equal to $0.2\xi_{S}$ , $\xi_{S}$ , and $2\xi_{S}$ . We have chosen such a relation between the thicknesses of the ferromagnetic layers to allow for independent remagnetization between the ferromagnetic layers F_1_ and F_2_ in the pseudo-spin-valve structure, which is consistent with experimental data for SF multilayer structures [8,16]. Such a choice preserves the difference in thickness between the F_1_ and F_2_ layers. In this case, the phase addition in the F_1_S_1_F_2_ part of the structure varies in the case of the parallel arrangement of magnetization vectors and remains constant in the AP case. The calculations for $\rho_{F}=2\rho_{S}$ , $\xi_{F}=2\xi_{S}$ , and $\rho_{F}=\rho_{S}$ , $\xi_{F}=\xi_{S}$ are shown in Figure 5. All other parameters have the same values as those used in Section 3.

It can be seen that increasing the thickness of the S_1_ layer leads to a suppression of the maximum value of $\delta^{max}$ and a shift in the position of the maximum to the larger $d_{F1}/\xi_{S}$ ratio. This behavior of the $\delta^{max}\left(d_{S1}\right)$ dependence is quite natural.

At small and fixed thicknesses of the F-films, an increase in $d_{S1}$ should be accompanied by a decrease in the influence of the F_2_ layer on the amplitude of the anomalous functions at the SF_1_ interface and a leveling of the difference between their values in the P and AP configurations. This leads to a shift in $d_{cr}^{AP}$ to larger values, to a convergence of $d_{cr}^{AP}$ and $d_{cr}^{P}$ , and to a suppression of $\delta^{max}$ with increasing $d_{S1}$ . This suppression is clearly seen in Figure 5a,b. It actually means that the growth of $d_{S1}$ leads to the splitting of the SF_1_S_1_F_2_s structure into two weakly interacting SF_1_S_1_ and S_1_F_2_s blocks. With the thicknesses of their superconductors several times larger than $\xi_{S}$ , their own superconductivity is sufficient to synchronize the phases of the order parameter and the anomalous functions as the spatial coordinate moves away from the SF boundaries. In this limit, the parameter $\delta^{max}\to 0$ , and the values of $\Delta_{s}$ in P and AP configurations can only differ in sign.

For large and fixed values of $d_{F1}$ , superconductivity in the vicinity of SF interfaces is strongly suppressed in the first approximation. It is obvious that the thicker the S_1_ interlayer, the faster the recovery of superconductivity in the AP case compared to the P case. This is why the parameter $\delta^{max}$ appears larger as $d_{S1}$ becomes thicker.

In an intermediate segment of $d_{F1}$ , the functions $\delta^{max}\left(d_{F1}\right)$ reach the maximum. The position of the maximum on the $d_{F1}$ scale is shifted to the larger $d_{F1}$ with increasing $d_{S1}$ . This tendency is quite obvious. The ferromagnetic layers F1 and F2 are the cause of the rotation of the pairing phase Θ(F) . The changes in Θ(F) are simply additive in the case of the zero thickness of the S1-layer. At the same time, the superconducting order in the S1-layer tends to return the phase to 0 and has a negative effect on the overall phase rotation. The larger the regions occupied by superconductors, the thicker the F-layer should be, which controls the final state of the SF_1_S_1_F_2_s structure.

Figure 5c shows that there is no shift in the position of $\delta^{max}\left(d_{F1}\right)$ as $d_{S1}$ increases in the case of the substitution of the S_1_-film by a normal metal. Due to the substitution, the regions occupied by superconductors do not change as $d_{S1}$ increases. As a result, there is no shift in the maximum in the $\delta^{max}\left(d_{F1}\right)$ dependencies.

The color palette in Figure 5d gives the value of $\delta^{max}$ as a function of the $d_{S1}/\xi_{S}$ and $d_{F1}/\xi_{S}$ ratios. The red color corresponds to the maximum values of $\delta^{max}$ . The blue color corresponds to the minimum values. The data presented in Figure 5d allow us to determine, for fixed values of $d_{S1}/\xi_{S}$ , which thickness of the F-layer should be chosen in order to realize states having the maximum value of $\delta^{max}$ and a positive or negative value of $\Delta_{s}\left(d_{s}\right)$ . For convenience, all values of $d_{s}^{max}$ at which $\delta^{max}$ is reached are not shown in Figure 5, as they have no additional meaning.

**Figure 6:**
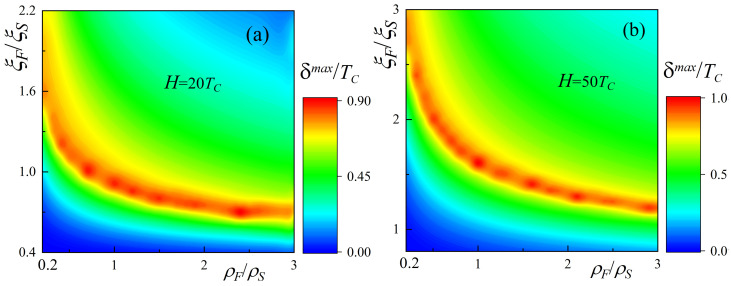
Maps for material parameters of ferromagnets $\rho_{F}$ , $\xi_{F}$ for the maximum difference in magnetization reversal $\delta^{max}$ at thick exchange energies $H=20T_{C}$ (**a**) and $H=50T_{C}$ (**b**). Other parameters of the SF_1_S_1_F_2_s structure are $d_{S}=5\xi_{S}$ , $d_{F}=0.15\xi_{S}$ , $d_{S_{1}}=0.2\xi_{S}$ , $d_{F_{1}}=0.25\xi_{S}$ , $T=0.5T_{C}$ , $\gamma_{B}=0.3$ .

Finally, we have studied the influence of the exchange energy of ferromagnets on the triggering parameters. Figure 6 shows maps of $\delta^{max}$ versus the material parameters $\rho_{F}/\rho_{S}$ and $\xi_{F}/\xi_{S}$ for the values of $H=20T_{C}$ (a) and $H=50T_{C}$ (b), which are weaker compared to the $H=100T_{C}$ that we used previously to obtain Figure 4a. It can be seen that the general form of the dependencies has been preserved. As in Figure 4a, there are two regions of parameters that divide the $\rho_{F}/\rho_{S}$ and $\xi_{F}/\xi_{S}$ planes into two regions that differ in the sign of $\Delta_{s}$ . The absolute values of $\delta^{max}$ are rather weakly dependent on *h*. At the same time, the position of the high-TE region is significantly shifted with the change in H. While for strong ferromagnets $H=100T_{C}$ , the significant TE effect appears only at $\xi_{F}\geq 2\xi_{S}$ and makes significant demands on the choice of materials, at $H=20T_{C}$ , the strongest TE effect is available in the interval $0.5\xi_{S}\leq \xi_{F}\leq 1.5\xi_{S}$ , which is quite reasonable for experimental realization. For convenience, all values of $d_{s}^{max}$ where $\delta^{max}$ is reached are not shown in Figure 6, as they are similar to those in Figure 4b.

## 5. Discussion and Conclusions

Our studies of the trigger effect in the SF_1_S_1_F_2_s structure have shown that it is very stable with respect to variations in its material and geometrical factors. The effect itself is that the SF_1_S_1_F_2_ spin valve does not control the superconducting state of the whole structure, but only that of its F_2_s part. In this case, the fact that the F_2_s block is in the pre-critical state is significantly exploited. The critical thickness of the s-film is determined from the equality of the order parameter and the anomalous Green functions at the F_2_s boundary to zero and the equality of the normal derivative to zero at the free boundary of the superconductor. It lies in the neighborhood of about $3.5\xi_{S}$ . For such large values of the critical thickness, the order parameter and the anomalous Green functions have the opportunity to increase from zero to values comparable to $T_{C}$ at the free surface of a superconductor with the increasing spatial coordinate. We have shown that in SF_1_S_1_F_2_s devices, there is a large difference in the critical thickness of their F_2_s part between parallel and antiparallel F-film magnetization vector orientations. This difference is the basis of the trigger effect we discovered. It allows the superconductivity in the s-film of the structure to be switched on or off by changing the mutual orientation of the vectors $M_{1,2}$ . Moreover, with such a switch, the absolute values of the order parameter at the free surface of the s-layer can differ only slightly from its equilibrium values. Importantly, the sign of the order parameter can be either positive or negative. This opens up new possibilities for the design of devices to control the inductance and critical current of Josephson junctions.

For example, the critical current of tunneling SF_1_S_1_F_2_sIS structures, where “I” denotes a layer with tunnel-type conductivity, is determined by the superconducting material parameters of those regions of the s- and S-films that are adjacent to the I-layer. By exploiting the trigger effect in the SF_1_S_1_F_2_s electrode of the SF_1_S_1_F_2_sIS structure, one can provide its switching between 0 and π states, while its critical current and characteristic voltage would be close to thsoe of standard junctions in digital circuits.

At the same time, the lack of phase synchronization of the order parameter and anomalous Green’s functions on the free surface of the s-layer is an important feature that should be taken into account in the design of Josephson tunnel structures that use the trigger effect in their operation. As a consequence of this desynchronization, it is impossible to determine the phase difference in the order parameters between the s- and S-electrodes, which determines the current–phase relationship of the SF_1_S_1_F_2_sIS structure. A similar situation takes place in SNS sandwiches [53] and variable-thickness bridges [54] and is solved by maintaining a global phase difference, the role of which in SF_1_S_1_F_2_sIS contacts should be taken over by the phase difference of order parameters of their massive S-electrodes, determined at their free boundaries. Physically, this means that the role of the weak region in SF_1_S_1_F_2_sIS Josephson junctions is played not by the insulating layer but by the whole SF_1_S_1_F_2_sI region, including the part of the massive S-electrode, which borders the F1-film. The determination of the current–phase relation and the operating modes of SF_1_S_1_F_2_sIS spin valves will be published elsewhere.

## Data Availability

The data presented in this study are available on request from the corresponding author.
